# Microbead-based extracorporeal immuno-affinity virus capture: a feasibility study to address the SARS-CoV-2 pandemic

**DOI:** 10.1007/s00604-023-05671-9

**Published:** 2023-02-18

**Authors:** Gabor Jarvas, Dora Szerenyi, Hajnalka Jankovics, Ferenc Vonderviszt, Jozsef Tovari, Laszlo Takacs, Fanni Foldes, Balazs Somogyi, Ferenc Jakab, Andras Guttman

**Affiliations:** 1 Research Institute of Biomolecular and Chemical Engineering, Faculty of Engineering, University of Pannonia, Veszprem, Hungary; 2 Bio-Nanosystems Laboratory, Research Institute of Biomolecular and Chemical Engineering, Faculty of Engineering, University of Pannonia, Veszprem, Hungary; 3 Department of Experimental Pharmacology, National Institute of Oncology, Budapest, Hungary; 4 Laboratory of Monoclonal Antibody Proteomics, Department of Human Genetics, Faculty of Medicine, University of Debrecen, Debrecen, Hungary; 5 National Virology Laboratory, BSL-4 Laboratory, Szentagothai Research Centre, University of Pecs, Pecs, Hungary; 6 Institute of Biology, Faculty of Sciences, University of Pecs, Pecs, Hungary

**Keywords:** Immuno-affinity, Extracorporeal treatment, Virus capture, SARS-CoV-2, Viremia, Hemoperfusion, Glass microbeads

## Abstract

In this paper, we report on the utilization of micro-technology based tools to fight viral infections. Inspired by various hemoperfusion and immune-affinity capture systems, a blood virus depletion device has been developed that offers highly efficient capture and removal of the targeted virus from the circulation, thus decreasing virus load. Single-domain antibodies against the Wuhan (VHH-72) virus strain produced by recombinant DNA technology were immobilized on the surface of glass micro-beads, which were then utilized as stationary phase. For feasibility testing, the virus suspension was flown through the prototype immune-affinity device that captured the viruses and the filtered media left the column. The feasibility test of the proposed technology was performed in a Biosafety Level 4 classified laboratory using the Wuhan SARS-CoV-2 strain. The laboratory scale device actually captured 120,000 virus particles from the culture media circulation proving the feasibility of the suggested technology. This performance has an estimated capture ability of 15 million virus particles by using the therapeutic size column design, representing three times over-engineering with the assumption of 5 million genomic virus copies in an average viremic patient. Our results suggested that this new therapeutic virus capture device could significantly lower virus load thus preventing the development of more severe COVID-19 cases and consequently reducing mortality rate.

**Graphical Abstract:**

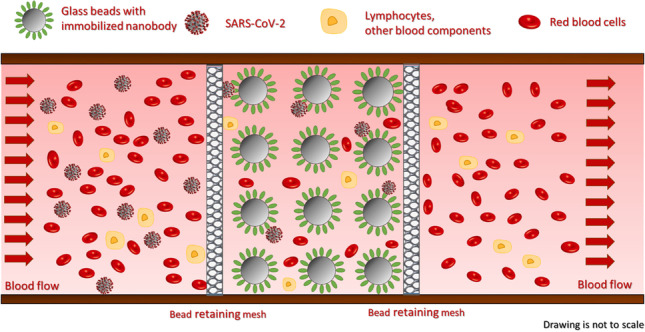

**Supplementary Information:**

The online version contains supplementary material available at 10.1007/s00604-023-05671-9.

## Introduction

Since the first hospitalizations in December 2019 [1], and the detection of the new coronavirus strain in January 2020 [2], the COVID-19 outbreak causes public healthcare and social crisis worldwide [3], and apparently remains for years [4]. During the first year of this pandemic, almost 100 million people got infected [5]. PCR methods and lateral flow tests are the most widely used diagnostic tools [6, 7], but CRISPR/CAS systems [8], antibody functionalized bioactive nanomaterials, and other technologies were also implemented to overcome this unseen global challenge [9–11]. Parallel to the diagnostic endeavors, new therapy strategies have been utilized and/or developed, such as antivirals, convalescent plasma treatments, antibody therapies, extracorporeal treatments, and virus depletion methods for clinical use. Furthermore, vaccine development against severe acute respiratory syndrome coronavirus 2 (SARS-CoV-2) was immensely accelerated [12] and as of the beginning of 2022, vaccination seems to be an effective way of immunization [13–18]. However, naturally occurring genetic viral mutations resulted in different variants prolonging the challenge [18].

Approximately 17–40% of SARS-CoV-2 positive patients reported no typical symptoms [19, 20] while on the other hand, about 15–30% of patients develop respiratory failure requiring hospital admission [21], 12% need ventilation, and 3% extracorporeal life support [14, 22]. Certain comorbidities such as hypertension, diabetes, and obesity are reportedly represent serious risk factors with poor prognosis [23–25]. Cardiovascular complications including thrombosis [26] and systemic, cytokine-mediated inflammation are also associated with severe SARS-CoV-2 infection. Most importantly, high viral load during viremia predicts poor survival [27–29]. Later in the disease course, approximately 2 weeks after the onset of the symptoms and initial viral replication in the upper respiratory tract, spreading progresses towards the lower respiratory tract and creates secondary viremia followed by an attack against ACE2-expressing tissues [30]. Spreading correlates with clinical deterioration, which usually occurs roughly 2 weeks after the onset of the symptoms. In addition, other organs possibly reached directly by the virus through the circulation during persisting or temporary viremia may also occur in severe COVID-19 cases [31–33]. High plasma viral loads indicate increased severity, potential organ damage, and high chance of mortality [27, 30, 34–36].

In general, separation and purification technologies are frequently studied and utilized for biomedical and clinical applications [37–45]. Extracorporeal membrane oxygenation and extracorporeal CO_2_ removal can be used to address respiratory failures. Furthermore, cytokine removal by continuous renal replacement therapy or direct hemoperfusion with specific filters, HA resin hemoperfusion cartridges, and Toraymixin polymixin-B endotoxin removal are available therapeutic strategies during cytokine storm caused by SARS-CoV-2 infection [13, 46, 47]. Early cytokine removal may prevent the progression of respiratory failure or other organ dysfunctions in critically ill patients [48, 49], extracorporeal treatments, applied in clinical practice in the case of COVID-19 intensive care units, are based on non-specific binding or the adsorption of molecules to a large specific surface area having sponge-like symmetric pore structure [47, 50–52].

Next to the hemofilters, hemoperfusion and plasmapheresis procedures, such as heparin-immobilized polyethylene beads, are also used for non-specific pathogen removal from the bloodstream. The attached heparin binds viruses, bacteria, fungi, and toxins similarly to heparane-sulfate interacting with cell surface [53, 54]. Hemopurifier lectin affinity plasmapheresis filters have also been designed for whole virus, exosome, and exosomal microRNA removal and investigated for COVID-19 therapy [55].

In this paper, we report a feasibility study for the development and implementation of a SARS-CoV-2 capture device to specifically remove virions from the circulation, preventing the development of serious clinical outcomes. The approach combines the specificity of affinity chromatography with the high throughput hemoperfusion technique to fight the coronavirus pandemic. A laboratory scale hemoperfusion system was designed and fabricated to study the feasibility of the proposed approach. The setup has been tested in a BSL-4 laboratory environment with live virus suspensions as representative conditions and models to explore any potential risk factors before further development.

## Materials and methods

### Chemicals

Picoline borane, DMEM cell culture media, and phosphate buffered saline (PBS, pH 7.4) were from Sigma-Aldrich (St Louis, MO). Polyethylene-glycol was purchased from Merck (Kenilworth, NJ). Fetal bovine serum, trypsin-EDTA, and 3-aminopropyltriethoxysilane (APTES) were from Thermo Fisher Scientific (Waltham, MA). Glutaraldehyde was purchased from Carl Roth Chemicals (Karlsruhe, Germany). Methanol and absolute ethanol were purchased from VWR (Radnor, PA). Hydrochloric acid, sulfuric acid, and dry toluene were purchased from Molar Chemicals Kft. (Halasztelek, Hungary). Kanamycin was from SERVA Electrophoresis GmbH (Heidelberg, Germany). Isopropyl β-D-1-thiogalactopyranoside (IPTG) was from Biosynth AG (Staad, Switzerland). The SDS PAGE gel was made from 40% acrylamide solution from Bio-Rad (Hercules, CA) and HPLC grade water in a ratio of 37.5:1. Buffer “A” contained NaH_2_PO_4_ from Merck (Kenilworth, NJ) and NaCl from VWR (Radnor, PA). Imidazole for buffer “B” was purchased from Merck.

### Expression and purification of SARS-CoV-2 spike protein–specific single-domain antibody

The coding sequence of the publicly available single-domain antibody (sdAb) against SARS-CoV-2 spike protein was codon optimized for *E. coli* [56] and the protein expressed from plasmid pET28b in SHuffle T7 *E. coli* strain, followed by purification using Ni-affinity chromatography as published earlier [57, 58]. The details of the procedure can be found in the Electronic Supporting Material.

### Immobilization of anti-SARS-CoV-2 sdAb onto the surface of glass beads

It should be noted that increased stress level of >150 Pa may cause extensive cell damage. To avoid any damage of the different blood constituents, a hydro-dynamically similar model system to an approved extracorporeal technology, i.e., Cytosorb (Berlin, Germany), was utilized. For this purpose, commercially available monodisperse 450 μm diameter soda-lime glass beads were obtained from Glass Sphere (Jablonec nad Nisou, Czech Republic) due to their available immobilization chemistry and biocompatibility (composition: SiO2 61–67%, Na2O 10–18%, CaO 5–10%, B2O3 1–5%, Al2O3 3–8%). The SARS-CoV-2 spike protein–specific sdAb was immobilized onto the surface of the beads as follows. First, the beads were treated with a 1:1 mixture of cc. hydrochloric acid and methanol for 30 min at room temperature, followed by washing with HPLC grade water. After the washing step, the beads were treated with cc. sulfuric acid for 30 min at room temperature. The beads were washed and treated by boiling HPLC grade water for 30 min [59] and dried in an oven at 100 °C for 40 min. The dried beads were shaken in 3% APTES in dry toluene for 2 h at room temperature. Then, the excess reagents were rinsed off by dry toluene and the beads were treated at 100 °C until complete drying. The beads were then shaken in 2% glutaraldehyde in HPLC water for 1 h at room temperature and the excess glutaraldehyde was removed by rinsing with HPLC grade water. 1 mg/ml picoline borane solution was prepared in 5% ethanol in HPLC water as coupling buffer, and then 100 μl of 80 μg/ml sdAb solution was added. The beads were shaken overnight in the coupling buffer at 4 °C followed by rinsing with PBS, and stored at 4 °C until further processing. Non-specific binding sites were blocked by incubating the beads in 10 mg/ml polyethylene-glycol (MW 8000) containing PBS at 4°C for four hours before use.

### Virus propagation

SARS-CoV-2 (hCoV-19 / Hungary / SRC_isolate_2 / 2020, Accession ID: EPI_ISL_483637) virus isolate was used for the experiments. Propagation of the viruses was carried out in VeroE6 cells (African green monkey kidney epithelial, ATCC CRL-1586) cultured in DMEM cell culture media containing 10% heat-inactivated fetal bovine serum. Cells were incubated at 37 °C in humidified air supplied with 5% CO_2_.

### Virus capture

All virus culturing and capture experiments were carried out in the facility of the National Virology Laboratory, University of Pecs, Hungary, which is a Biosafety Level 4 (BSL-4) facility fulfilling all the requirements of the US Centers for Disease Control and Prevention guide for Biosafety in Microbiological and Biomedical Laboratories (6^th^ Edition) [60]. Shortly, the laboratory is located in a separate building having dedicated supply and exhaust ventilation equipment with vacuum and decontamination systems. Operators were wearing a full-body, air-supplied positive pressure suit, while −120 Pa pressure was maintained in the rooms. The utilized conditions aimed to prevent any aerosol mediated infections.

**Fig. 1:**
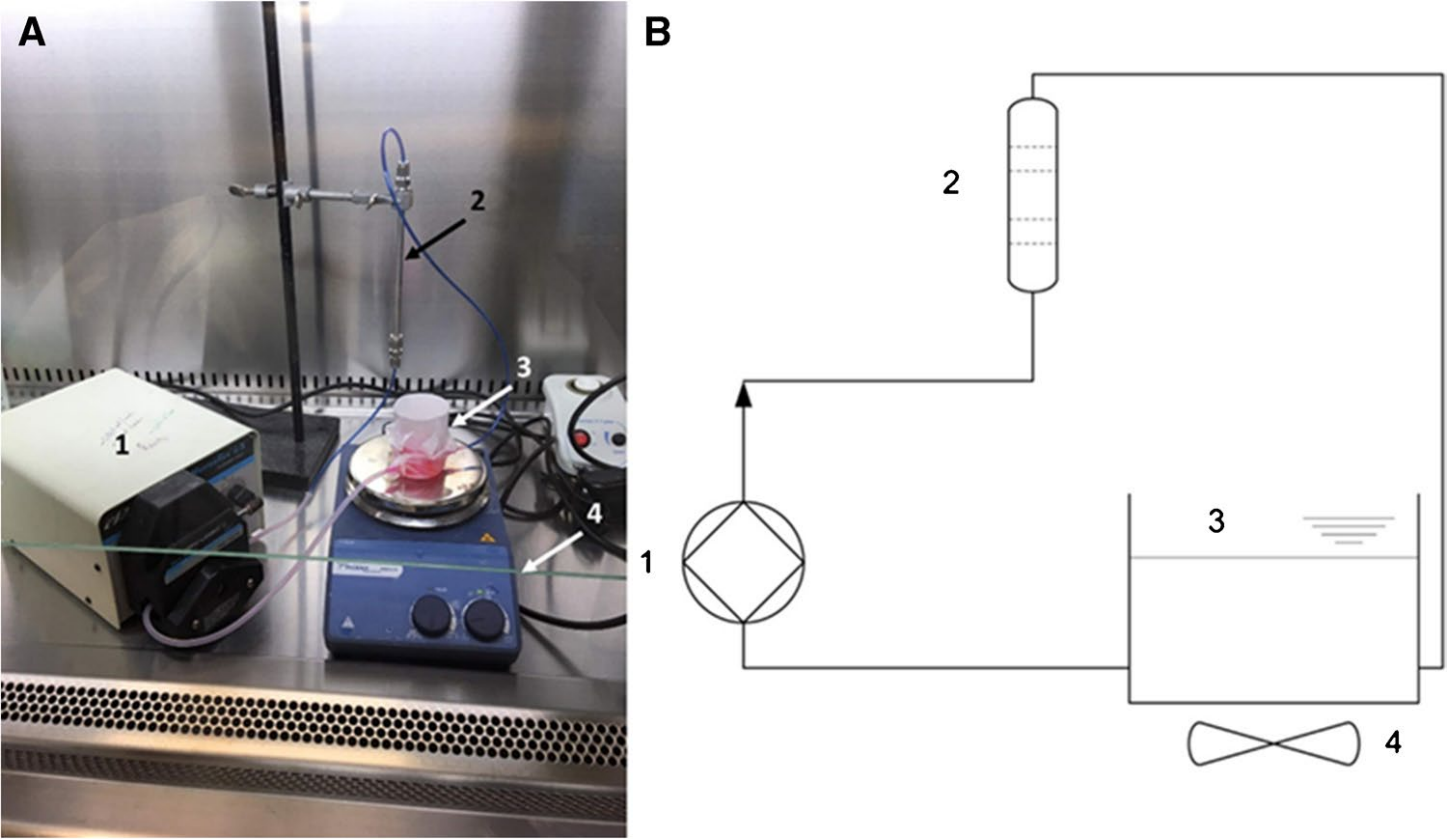
The laboratory scale system with the test cartridge (panel **A**) and its schematic representation (panel **B**). The circulation was supported by a peristaltic pump (1). The parts of the system—cartridge (2), vessel (3)—were connected with polyethylene tubing. The virus suspension in the vessel was homogenized by a magnetic stirrer (4). The vessel served as sampling point for the initial and residual samples

Standard stainless steel HPLC columns (length: 16.5 cm, I.D. 4.6 mm) were modified to obtain laboratory-scale test cartridges. Four hundred micrometer hole size meshes were placed in the Swagelok fittings to retain the beads during the circulation. Four grams of anti-SARS-CoV-2 sdAb immobilized beads was loaded into the cartridges for the virus capture experiment. The viruses, at the average of ~350 viral genome copies/μl concentration, were spiked into 30 ml DMEM medium and the virus suspension was circulated through the cartridges under investigation using a Masterflex peristaltic pump (Cole-Parmer, Vernon Hills, IL) for 2 h (i.e., 20 cycles in total). An open-lid vessel was inserted in the circulation system to equilibrate the fluid level and ensure bubble-free flow. Furthermore, the vessel served as injection and sampling point as well. The schematics and photo of the experimental setup are shown in Fig. 1. The virus suspension was continuously homogenized during the experiments using a magnetic stirrer. The flow rate was set to 5 ml/min (corresponding to 155 cm/min linear velocity). To monitor any non-specific binding of the virus particles, a control experiment was also performed. The setup of the control experiment was identical as described above, but only the linker (i.e., glutaraldehyde) was immobilized onto the bead surface, and the non-specific binding sites were masked by 10 mg/ml polyethylene-glycol (MW 8000) containing PBS as suggested by Monard et al. [50]. The virus suspension was circulated continuously for 2 h and after the circulation was stopped, the column was rinsed with PBS and the beads were transferred to falcon tubes. Virus particles were detached from the surface of the beads by washing with 1 ml 1x trypsin-EDTA solution in PBS for 5 min. Then, 2 ml PBS was added before further incubation for 5 min at 37°C. The bead-virus particle suspension was vortexed thoroughly, then the beads were allowed to sink and the supernatant was sampled (referred to as eluent).

### Quantitative virus genome copy determination

Duplicate samples of 100 μl were taken from the vessel (referred to as initial and residual) as well as from the supernatants (referred to as eluent) to determine the initial virus concentration and the number of captured virion particles, respectively. The number of captured virus particles was quantitatively determined by ddPCR (QX200 Droplet Digital PCR, Bio-Rad) as published earlier [61, 62]. Shortly, ddPCR is based on water-oil emulsion droplet technology. A sample is fractionated into a certain number of droplets, and PCR amplification of the template molecules occurs in each individual droplet [63]. After PCR amplification, concentrations are determined using the Poisson distribution of fluorescent and non-fluorescent droplets [64]. The Bio-Rad QuantaSoft software was used to evaluate the results. Nucleic acid isolation from the samples was performed using a Monarch Total RNA Miniprep Kit (New England Biolabs, Ipswich, MA) according to the vendor’s instruction to quantify the viral RNA. Furthermore, conventional qPCR method was utilized for the detection of SARS-CoV-2 virions in the eluent [65].

### Scanning electron microscopy

Captured virus particles were visualized using scanning electron microscopy (FEI/ThermoFisher Apreo S LoVac). Observation by STEM was carried out in transmission mode under high-vacuum with 30 kV accelerating voltage. Samples were observed without fixation by following a standard air-drying procedure [66]. UV-C inactivated virus stock was purchased from RoLink Biotechnology (Pecs, Hungary). First, the virus suspension was dried onto a carbon-stabilized formvar-coated side-grid (SFR, Toronto, Canada) at room temperature for 20 min to determine its morphology. Then, glass beads with captured virus particles on their surface were taken out from the stainless steel cartridge after a regular capture cycle. To remove any remaining residues from the culture media, beads were rinsed with HPLC grade water. Low-vacuum mode was used with 10 kV accelerating voltage to detect the virus on the surface of the glass beads.

## Results and discussion

**Fig. 2:**
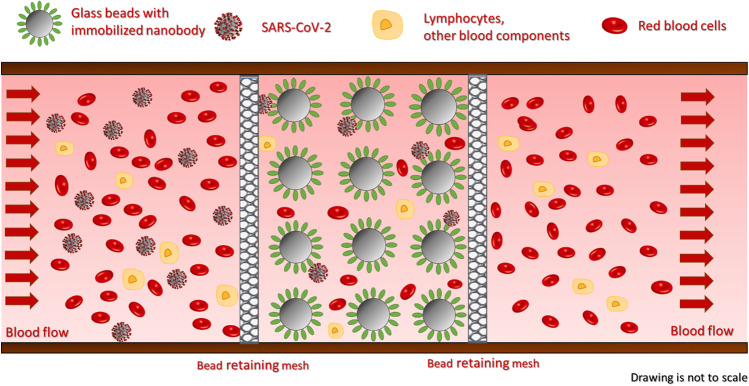
Schematic representation of the virus capture mechanism. The immobilized nanobodies specifically bound the spike protein of SARS-CoV-2 particles thus selectively capture them from the blood stream. Peripheral blood flows through the microbead packed bed from left to right. The bed is retained by the mesh. All intact blood components can be returned to the circulation without any known and/or expected side effects

In this paper, we report a feasibility study, first approach towards implementing a novel method to fight SARS-CoV-2 pandemic. A high throughput extracorporeal device has been designed to specifically remove virions from the circulation with the goal to avoid serious clinical outcome. A laboratory scale hemoperfusion system has been fabricated to study the feasibility of the proposed approach, which combined the specificity of affinity chromatography with the high throughput of hemoperfusion. The scheme of the proposed virus capture technique is shown in Fig. 2.

Capture efficiency was investigated by using an actual strain of SARS-CoV-2 in a BSL-4 classified laboratory. In the presented proof of concept experiments, the virus particles were captured from Dulbecco Modified Eagle Medium containing L-glutamine, sodium pyruvate, sodium bicarbonate, and phenol red indicator as described in the experimental section. Albeit, its complexity lags behind that of blood complexity, it can adequately model the virus capture efficiency from fluidic circulation. Virus concentrations were determined by ddPCR, both in the active and control experiments (Table 1).

**Table 1:** Results of the ddPCR analysis. The reported virus copy values represent the genomic concentrations. SARS-CoV-2 virus particles were spiked into PBS buffer, followed by virus capture utilizing the technology proposed in this paper. All experiments were done in duplicates

		Initial	Residual	Eluent
		[copy/μl]		
Active	Sample 1	364	23	129
	Sample 2	331	29	117
	Average	348	26	123
Control	Sample 1	303	102	73
	Sample 2	397	108	93
	Average	350	105	83

As apparent from Table 1, some virus particles must have been non-specifically bound to the plastic components of the equipment (buffer vessel, tubing, and connectors) and on the surface of the glass beads, represented by the difference between the *initial*-*residual+eluent*. However, it should be emphasized that the surface-to-volume ratio of this laboratory scale experimental setup was significantly higher than that of in an actual hemoperfusion device; thus, non-specific binding was considered to be overrepresented. The measure of non-specific binding was determined by balancing the captured virus copies in the active and control experiments. Non-specific binding of virions or any proteinous macromolecules is governed by physical adsorption (or physisorption), in which van der Waals forces, hydrophobic interactions, electrostatic interactions, and hydrogen bonds play essential roles. Physisorption is resulted due to the interaction of electron configuration of the contacting atoms or molecules. In spite of the numerous technological advancements in the field of protein immobilization [67], non-specific binding remained a critical bottleneck due to the lack of fundamental understanding of the phenomenon. Consequently, non-specific binding could not be completely avoided only decreased; therefore, its relatively high apparent measure is not surprising [67, 68]. From the practical point of view, it is expected that non-specific binding will be less important in an actual clinical environment, as during the priming step (i.e., the process, which removes the storage solution and any air bubbles from the extracorporeal cartridge) human serum albumin will block most if not all non-specific binding sites.

Note, only those virus particles were involved in the evaluation, which remained in the circulation and did not bind non-specifically, i.e., not removed from the circulation. Considering the reported genomic virus concentrations (Table 1), the volume of the processed virus suspensions, the residual and the eluted samples (30 ml, 30 ml, and 3 ml, respectively), the following absolute virus copies were counted: 10,425,000 (initial); 778,500 (residual); and 369,000 (eluent) in the case of the active experiment and 10,500,000, 3,150,000, and 247,900 in the case of the control. Thus, as a first approximation, we can conclude that the active cartridge removed 32% of the virus particles from the circulation, while the control cartridge was able to adsorb only 7%, apparently representing the level of non-specific binding. In other words, as both the active and control experiments also captured virions non-specifically; thus, only their differences were considered later as immuno-affinity captured virus particles, not the absolute values. The 4–5-fold difference in the residual virion copy number between the active and control experiments is notable, therefore suggests the feasibility of the proposed technology.

**Fig. 3:**
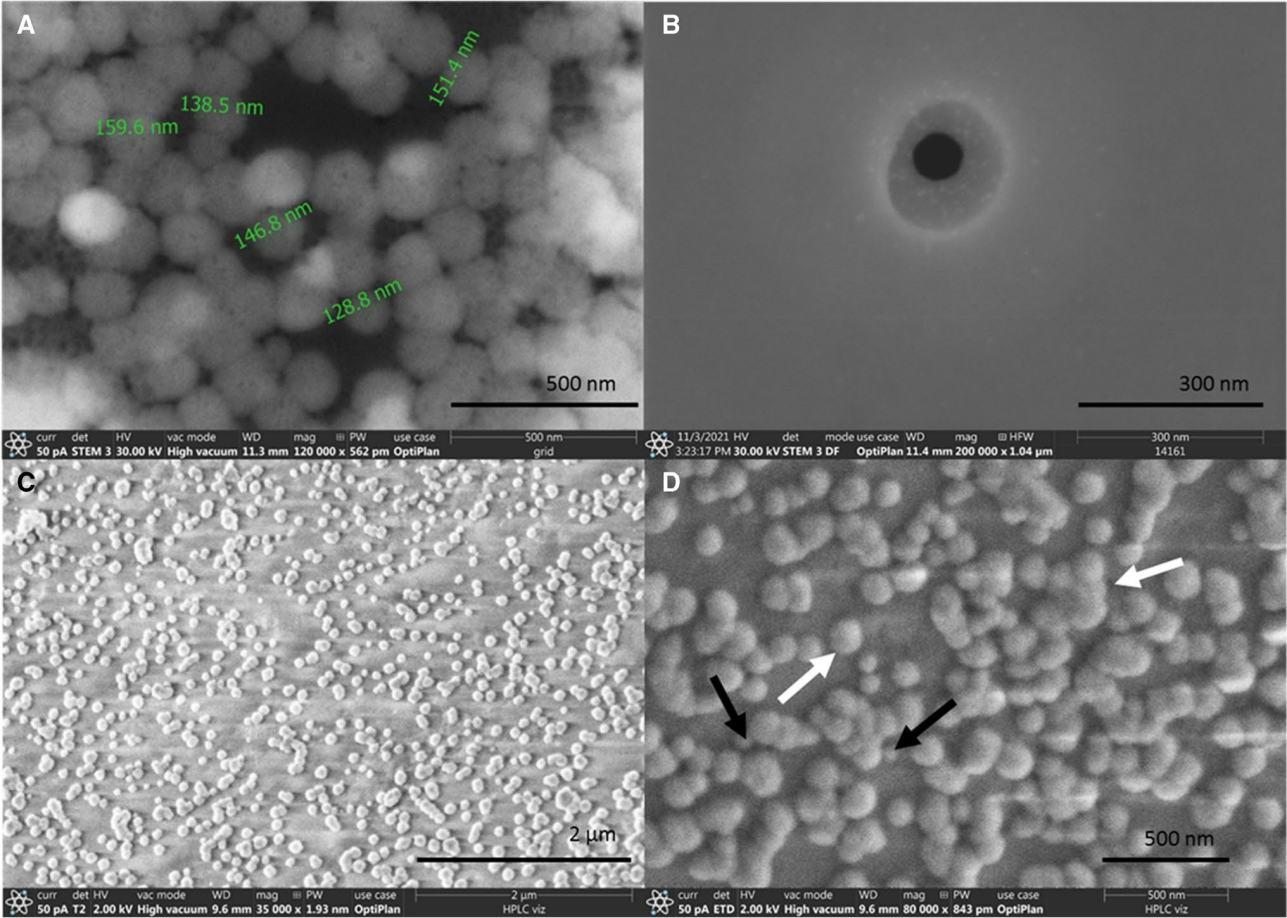
Scanning electron microscope (SEM) images of the virions. Panel (**A**): virus suspension dried onto a microscope slide-grid. Panel (**B**): single virion; the black patch visible in the center of the virus particle was caused by the electron beam. Panels (**C**) and (**D**): captured virus particles on the surface of the beads. Single and aggregated virions are indicated by black and white arrows, respectively. Aggregation of virus particles occurred due to the drying step of the sample preparation at room temperature

After the capture efficiency studies, scanning electron microscope (SEM) imaging was utilized to visualize the captured viruses on the surface of the beads. To this end, ~4·10^5^ PFU/ml concentration virus suspension was dried onto a microscope slide-grid to observe certain morphologies of the virions under the conditions of the actual experimental setup (see Fig. 3A and B). Figure 3C and D show the captured virus particles on the surface of the beads. Please note that here the same capturing method was used as for the efficiency measurements above, but the captured virus particles were rinsed with HPLC grade water to remove any residual PBS in order to improve image sharpness. The resulted SEM images are apparently similar to recently published pictures made under similar conditions [69, 70]. After SEM imaging, the beads were trypsin-EDTA washed for verifying the microscopy observations. The existence of SARS-CoV-2 virions in the eluent was successfully verified by conventional qPCR method.

According to the current clinical protocols, similar extracorporeal treatments may last up to 24 h [71], that, based on our results, would readily capture practically all circulating virus particles. Both during the active and control experiments, the captured virus particles were eluted from the surface of the glass beads by buffer rinse with trypsin wash. Comparing the active and control experiments with the down-scaled laboratory model device filled with 2.6 cm^3^ of glass beads, the difference in the number of specifically captured viruses was considered to be the actual performance, i.e., found to be 120,000 virus copies. The captured 120,000 virus particles from  the virus culture media circulation, suggested the partitioning ability of 15 million virus particles with an actual therapeutic size column. In other words, since the actual size of an average hemofiltration cartridge is about 330 cm^3^, the virus-binding capacity of our design is expected to be around 15 million. There is a reported correlation between human plasma SARS-CoV-2 virus concentration and COVID-19 disease course and mortality [27, 72]. Viremia is observed in 100%, 52.6%, and 11.1% of intensive care unit, non-intensive care unit, and outpatients of COVID-19 patients, respectively, and typical virus concentrations in the plasma of viremic patients range from 100 to 1000 copies/ml [73, 74]. Considering that an adult has an average of 5 l of blood, the amount of SARS-CoV-2 to be captured from the bloodstream at one time is expected to be around 5 million virus particles, so the system design reported in this paper holds the promise to have threefold excess capture capacity. Please note that the reported virus copy numbers in the literature represent the genomic concentrations, i.e., albeit the exact relationship between genomic virus concentration and infectious viral load level is still under debate [56, 75, 76]; all reported findings in this study should be interpreted from the technical feasibility point of view only. The actual therapeutic benefit of the proposed approach cannot be evaluated without comprehensive clinical trials. However, a very recent large cohort study indicated that the odds of mortality increased by 40% by each viremic day [77].

Please note the importance of future biocompatibility testing to shed light on any potential drawbacks of the proposed technology, which was not possible at this very early stage of the development. However, it is suspected that shear stress on blood elements, extracorporeal treatment–induced hypothermia, and blood exposure to non-biological surfaces may initiate perioperative systemic inflammatory response syndrome [78]. Another limitation of the method is the relatively fast appearance of various new mutated virus strains due to positive evolutionary selection, i.e., decreasing immune-affinity strength. In the presented particular case, the mutations resulted in the structure variation of the spike protein, which is targeted by the utilized sdAb for immune-affinity virus capture. Such mutations are very difficult to rapidly address from regulatory point of view, i.e., new antibody approvals, unless agencies authorize emergency use in case of urgent needs.

## Conclusions

A laboratory scale virus capture device was designed and fabricated to evaluate the feasibility of a novel immuno-affinity capture–based extracorporeal treatment approach. Due to the recent SARS-CoV-2 pandemic, its Wuhan (VHH-72) virus strain was chosen as model system to probe the proposed technology. Single-domain antibodies were produced by recombinant DNA technology and immobilized on the surface of glass micro-beads. A laboratory size, i.e., down-scaled model device, was utilized for the feasibility tests. The experiments conducted in BSL4 laboratory environment using live virus suspensions successfully proved the virus capture ability of the reported method. In the lack of effective antiviral drugs, especially during the outbreak of a rear or unknown virus caused pandemic, the suggested technology may serve as the base of an effective extracorporeal therapy, which helps patients to fight against the infection and significantly decrease the chance of being admitted to intensive care units. Furthermore, it is anticipated that after further development, the reported technology could possibly be an artificial organ platform, which represent an option to address organ failure issues and improve survival in case of viremia caused by several types of viral infections.

## Supplementary information

ESM 1 (DOCX 30 kb)
