# Supplementary material for: Microbead-based extracorporeal immuno-affinity virus capture: a feasibility study to address the SARS-CoV-2 pandemic
Source: Mikrochim Acta. 2023 Feb 18;190(3):95. doi: 10.1007/s00604-023-05671-9 (PMC9937867; doi:10.1007/s00604-023-05671-9)
Supplement: Supplementary file 1 — (DOCX 30 kb) [file 604_2023_5671_MOESM1_ESM.docx]

**Electronic Supplementary Material**

**Microbead based extracorporeal immuno-affinity virus capture: a feasibility study to address the SARS-CoV-2 pandemic**

Gabor Jarvas^1^, Dora Szerenyi^1^, Hajnalka Jankovics^2^, Ferenc Vonderviszt^2^, Jozsef Tovari^3^,

Laszlo Takacs^4^, Fanni Foldes^5,6^, Balazs Somogyi^5,6^, Ferenc Jakab^5,6^, and Andras Guttman^1*^

^1^Research Institute of Biomolecular and Chemical Engineering, Faculty of Engineering, University of Pannonia, Veszprem, Hungary;

^2^Bio-Nanosystems Laboratory, Research Institute of Biomolecular and Chemical Engineering, Faculty of Engineering, University of Pannonia, Veszprem, Hungary;

^3^Department of Experimental Pharmacology, National Institute of Oncology, Budapest, Hungary;

^4^Laboratory of Monoclonal Antibody Proteomics, Department of Human Genetics, Faculty of Medicine, University of Debrecen, Debrecen, Hungary;

^5^National Laboratory of Virology, BSL-4 Laboratory, Szentagothai Research Centre, University of Pecs, Pecs, Hungary;

^6^Institute of Biology, Faculty of Sciences, University of Pecs, Pecs, Hungary.

*corresponding author: Andras Guttman, e-mail: guttman@mik.uni-pannon.hu

***Expression and purification of SARS-CoV-2 spike protein specific* *single domain antibody***

The coding sequence of the publicly available single domain antibody (sdAb) against SARS-CoV-2 spike protein was codon optimized for *E. coli* [[1](#_ENREF_1)]:

(CAGGTGCAGCTGCAGGAAAGCGGCGGCGGCCTGGTGCAGGCGGGCGGCAGCCTGCGCCTGAGCTGCGCGGCGAGCGGCCGCACCTTTAGCGAATATGCGATGGGCTGGTTTCGCCAGGCGCCGGGCAAAGAACGCGAATTTGTGGCGACCATTAGCTGGAGCGGCGGCAGCACCTATTATACCGATAGCGTGAAAGGCCGCTTTACCATTAGCCGCGATAACGCGAAAAACACCGTGTATCTGCAGATGAACAGCCTGAAACCGGATGATACCGCGGTGTATTATTGCGCGGCGGCGGGCCTGGGCACCGTGGTGAGCGAATGGGATTATGATTATTATCTGGATTATTGGGGCCAGGGCACCCAGGTGACCGTGAGCAGC)

The genes with 5´-Ndel and Xhol cleavage sites were synthetized by Twist Bioscience (South San Francisco, CA) and cloned into a pET28b expression vector (Novagen, Darmstadt, Germany). Shuffle T7 Express E. coli (New England Biolabs, Ipswich, MA) cells were transformed with the plasmids, according to the supplier’s instructions. 5 mL LB pre-culture supplemented with 50 µg/mL kanamycine (Kan) was prepared as reported earlier [[2](#_ENREF_2), [3](#_ENREF_3)], and the resuspended cells were used to inoculate 1L LB/Kan. The cell culture was grown at 30°C with 140 rpm shaking in a baffled flask until the OD_600_ value reached the 0.6-0.8 range. Protein expression was induced by the addition of 0.4 mM isopropyl β-D-1-thiogalactopyranoside (IPTG) and incubated overnight. The cells were harvested by centrifugation at 6000x*g* for 30 min, then washed with buffer ‘A’ (20 mM NaH_2_PO_4_, 500 mM NaCl, pH 7.5). Cells were resuspended on ice in 10 ml buffer ‘A’ containing EDTA-free Mini Complete protease inhibitor (Roche, Basel, Switzerland) and disrupted by sonication (8 x 30 s, 40% amplitude, Omni Sonic Ruptor, Perkin Elmer, Waltham, MA). After centrifugation and filtration, the supernatant was loaded onto a Ni(II)-saturated, pre-equilibrated 5 ml HiTrap Chelating column (GE Healthcare, Chicago, IL) and purified using a linear gradient of 25-300 mM imidazole containing buffer (5-60% ‘B’ buffer, 11 column volume). The pure protein was eluted at 250 mM imidazole concentration and dialyzed. Purity of sdAb was confirmed on 15% SDS PAGE gels and protein concentration was calculated using the following parameters given by ProtParam [[2](#_ENREF_2)]: aSARS-6His, 16.2 kDa, 37,025 cm^-1^M^-1^. The application of the optimized production protocol resulted in a typical yield of pure anti-SARS of 5 mg/L culture.

***References***

1. Wrapp, D., et al., *Structural Basis for Potent Neutralization of Betacoronaviruses by Single-Domain Camelid Antibodies.* Cell, 2020. **181**(5): p. 1004-1015.e15.

2. Reider, B., et al., *Integrated workflow for urinary prostate specific antigen N-glycosylation analysis using sdAb partitioning and downstream capillary electrophoresis separation.* Analytica Chimica Acta, 2021. **1184**: p. 338892.

3. Meszaros, B., et al., *N-glycomic Analysis of Z(IgA1) Partitioned Serum and Salivary Immunoglobulin A by Capillary Electrophoresis.* Current Molecular Medicine, 2020. **20**(10): p. 781-788.
